# Newly emerging type B insulin resistance (TBIR) during treatment with eculizumab for AQP4-IgG-positive neuromyelitis optica spectrum disorder (NMOSD): fatal outcome

**DOI:** 10.1007/s00415-023-12071-9

**Published:** 2023-11-14

**Authors:** S. Doubrovinskaja, M. Korporal-Kuhnke, S. Jarius, J. Haas, B. Wildemann

**Affiliations:** 1 Department of Neurology, University Hospital Heidelberg, Heidelberg, Germany; 2 Molecular Neuroimmunology Group, Department of Neurology, University Hospital Heidelberg, Heidelberg, Germany

**Keywords:** Neuromyelitis optica spectrum disorders (NMOSD), Aquaporin-4 antibodies (AQP4-IgG), Type B insulin resistance (TBIR), Eculizumab, Ravulizumab, Infections, Sepsis, Pneumonia, Fatal outcome, Death

## Abstract

Aquaporin-4 immunoglobulin G (AQP4-IgG) antibody-positive neuromyelitis optica spectrum disorders (NMOSD) are frequently associated with other autoimmune disorders, including systemic lupus erythematosus (SLE). Eculizumab (ECU) is a highly effective long-term treatment for NMOSD. However, ECU is known to increase significantly the risk of infection with encapsulated bacteria and sepsis. Recently, increased insulin resistance (IR) in patients with NMOSD has been suggested. Type B IR (TBIR) is a rare autoimmune condition often accompanying or preceding SLE. TBIR has not yet been reported in NMOSD. Objective: To report an ECU-treated patient with AQP4-IgG-positive NMOSD who developed fatal septic complications after the emergence of TBIR. Methods: Description of the clinical course over a period of 8 years. Results: A female patient was diagnosed with NMOSD at the age of 16 years. A variety of disease-modifying drugs failed to achieve sufficient disease control, resulting in severe tetraparesis. Treatment with ECU was started 6 years after NMOSD diagnosis and stabilized the disease. The patient developed TBIR 8 months after initiation of ECU therapy. Following high-dose intravenous methylprednisolone therapy for a clinical relapse and three further courses of ECU, the patient was admitted with severe pneumonia caused by the encapsulated bacterium *Klebsiella pneumoniae* and hypoglycemia. Despite multimodal therapy, the patient died from sepsis-related multiorgan failure 18 months after initiation of ECU. Conclusions: TBIR should be considered as differential diagnosis in patients with NMOSD presenting with disturbed glucose metabolism, irrespective of the presence of SLE. More real-world data are needed on the risk/benefit ratio of ECU treatment in patients who have co-existing autoimmune comorbidities that may compromise immune function. Strategies to mitigate the risk of serious infection in patients treated with ECU are discussed.

Dear Sirs,

Neuromyelitis optica spectrum disorders (NMOSD) are rare yet often severe autoimmune neurological diseases causing predominantly myelitis and optic neuritis. NMOSD can result in permanent para-/tetraplegia or blindness, with women predominantly affected [3, 6, 8]. Immunoglobulin G (IgG) autoantibodies against the water channel protein aquaporin-4 (AQP4), first described in 2004 [10], contribute directly to the pathogenesis of NMOSD [11]. For many years, maintenance treatment of NMOSD relied on classic immunosuppression with drugs such as corticosteroids, azathioprine, and mycophenolate mofetil, or on B-cell depletion with rituximab [8], all used off-label. Recently, however, treatment with the complement component C5 antibody eculizumab, which inhibits the late stage of the complement cascade, has been approved. In 2019, a randomized controlled study showed a significantly lower risk of relapse in NMOSD patients treated with eculizumab than in those who received placebo [18].

Here, we describe the case of an eculizumab-treated patient who developed fatal infectious complications following the emergence of an autoimmune glucose metabolism disorder. This patient was diagnosed with AQP4-IgG-positive NMOSD according to the 2015 International Panel for NMO Diagnosis criteria [23] at the age of 16 years after an attack of unilateral optic neuritis followed 5 months later by an episode of brainstem encephalitis and myelitis. AQP4-IgG was detected by means of a cell-based assay [9] (maximum titer 1:10,000), and myelin oligodendrocyte glycoprotein-antibody associated encephalomyelitis was excluded [7]. Despite treatment with a variety of disease-modifying drugs, including intravenous immunoglobulins, azathioprine (AZA), mycophenolate mofetil (MMF), and rituximab (RTX), the patient experienced at least six more episodes of myelitis over the following 4 years, leaving her unable to walk. The course of disease was complicated by multiple infections, including sepsis with methicillin-resistant *Staphylococcus aureus* and upper respiratory tract infection with *Haemophilus influenzae* during treatment with MMF. Despite laboratory evidence of full B-cell depletion during treatment with RTX, the patient experienced three further episodes of myelitis, including a particularly severe attack of brainstem encephalitis and longitudinally extensive transverse myelitis extending from the medulla oblongata to the conus, leading to paresis of both arms in addition to the existing paraparesis.

Therefore, 6 years after the onset of NMOSD and following complete meningococcal vaccination, the immunosuppressive treatment was switched to monotherapy with intravenous infusions of eculizumab 1200 mg every 2 weeks after induction with 900 mg weekly for 4 weeks and 1200 mg in week 5. This resulted in stabilization of NMOSD, with no further NMOSD attacks in the following year. However, 8 months after initiation of treatment with eculizumab the patient had recurrent episodes of hyperglycemia (HbA1c up to 13%; negative GAD, islet cell, and IA2 antibodies), followed 4 months later by frequent postprandial hypoglycemia, which persisted after discontinuation of insulin. The findings of hyperinsulinemia (up to 587 mU/l [normal: 6–25]), pronounced insulin resistance (HOMA-IR up to 614 [normal: < 2.3], C peptide 10.6 ng/ml [normal: 0.7–2]), normal triglyceride levels, and hyperandrogenism with elevated testosterone levels resulting in hirsutism, amenorrhea, acne, and androgenetic alopecia led to the diagnosis of type B insulin resistance (TBIR). Screening for insulin receptor autoantibodies was negative, and the patient did not present acanthosis nigricans. Magnetic resonance imaging of the abdomen showed no evidence of insulinoma.

Sixteen months after initiation of eculizumab therapy, severe hypoglycemia required prolonged hospitalization, leading to interruption of eculizumab treatment for 6 weeks. Five weeks after the discontinuation of eculizumab therapy the patient experienced a further episode of myelitis, resulting in aggravation of her tetraparesis, which was treated with high-dose intravenous methylprednisolone (1g/day for 5 days). After three further courses of eculizumab, the patient was admitted to a department of internal medicine with severe hypoglycemia and pneumonia caused by *Klebsiella pneumoniae* and *Candida albicans*, subsequently resulting in sepsis. A single plasma exchange intended to treat TBIR did not lead to significant improvement in the patient’s glucose status. Despite various antibiotic and antimycotic therapies (including vancomycin, meropenem, and imipenem/cilastatin) and intensive care treatment, the patient died of multiorgan failure 5 weeks after re-admission and 18 months after initiation of eculizumab therapy.

Recently, higher blood insulin levels and lower insulin sensitivity in patients with AQP4-IgG-positive NMOSD were reported in a single cohort and hypothesized to be linked to increased interleukin-6 serum levels [13]. Hyperglycemia may also occur as an adverse effect of steroids, which are used both to treat NMOSD attacks (with or without oral tapering) and, occasionally, to prevent an attack. However, co-existence of NMOSD and TBIR has not been reported. Of interest, TBIR did not occur until 8 months after initiation of treatment with eculizumab in our patient. TBIR is characterized by abnormalities in glucose homeostasis, ranging from hyperglycemia to fasting hypoglycemia and in females is often accompanied by hyperandrogenism—a condition present in our patient—and by acanthosis nigricans [2]. TBIR is triggered by autoantibodies targeting insulin receptors in a subset of patients [14]. However, detection of autoantibodies is not mandatory for diagnosis, and screening for anti-insulin receptor antibodies may be negative [22], as was the case in our patient. Both NMOSD and TBIR are frequently associated with other autoimmune diseases, most often with systemic lupus erythematosus (SLE) [2]. TBIR may also be the first symptom of SLE. Our patient did not (yet) meet the diagnostic criteria for SLE, however, and no evidence of other coexisting autoimmune disorders was found. Patients with TBIR often present in a severe catabolic state with marked weight loss [22]. Our patient had lost 9 kg within the 2 months preceding the diagnosis of TBIR. She never regained her original weight, instead reaching a stable BMI of around 17.5 kg/m^2^, which likely contributed to her compromised immune status.

Treatment with eculizumab is usually tolerated well but is associated with a significantly increased risk of infection (including sepsis) with *Neisseria meningitidis* (2000 times more common than in the general population) and other encapsulated bacteria [1]. Meningococcal vaccination at least 2 weeks before the initiation of treatment with eculizumab is, therefore, mandatory [1]. Moreover, fungal infections, including candidiasis, may occur in patients treated with eculizumab [1, 12]. Pneumonia was a common adverse event in eculizumab clinical trials [1]. In our patient, the course of disease was in fact complicated by pulmonary infection with an encapsulated bacterium, *Klebsiella pneumoniae*, and *Candida albicans*, followed by sepsis. It is not unlikely that the eculizumab therapy contributed to the emergence of this patient’s life-threatening infection and finally the fatal sepsis. Other factors, in particular a severely compromised immune status resulting from a catabolic state due to autoimmune insulin resistance and hypoglycemia, together with concomitant immunosuppression by the corticosteroids given to treat the myelitis, may also have contributed to the development of severe infection.

This case exemplifies the risks associated with the use of eculizumab in NMOSD patients who have concomitant autoimmune diseases that can cause secondary immunodeficiency. Data on the safety of eculizumab in such patients are limited thus far, but further research is important considering that eculizumab is increasingly being used to treat AQP4-IgG-positive NMOSD and that NMOSD is frequently associated with other autoimmune diseases that may comprise immune function (most commonly SLE) [4, 20]. The frequency of fatal outcomes from eculizumab therapy for NMOSD in real-life settings is unknown. A recent manufacturer-funded Japanese surveillance study reported serious drug-related reactions in 10% of cases and drug-related infectious adverse events in 6% but no fatalities after a mean treatment period of 44.6 (± 23.7) weeks (as a limitation, only 71 of 147 patients enrolled in the study could be included in the safety analysis) [16]. By contrast, five of 55 patients (9%) died during treatment with eculizumab in a European cohort (median treatment time 14.6 months), four of them from sepsis [19]. In a large Japanese real-life study on 1055 patients treated with eculizumab (3977 patient-years) for paroxysmal nocturnal hemoglobinuria (PNH), atypical hemolytic uremic syndrome (aHUS), or generalized myasthenia gravis, 3.2% died from severe infection (including meningococcal sepsis in two) [17]. Overall, 40 (8.4%) of 479 infection-related severe adverse events were fatal in 34 patients. It would be of high clinical interest to know whether concomitant autoimmune diseases were present also in some of these patients.

Infection with *Klebsiella pneumoniae* seems to be rare among patients treated with eculizumab: In a recent survey of serious infections in patients with PNH and aHUS, this bacterium accounted for 3.3% of all eculizumab-associated infections [21]. *Candida* species were noted in 11% of all cases [21]. As a limitation, however, it is unknown whether the large group of cases (63%) with non-specified causal organism type in that survey included further cases of unrevealed *Klebsiella* or *Candida* infection.

Relatively little is known about specific risk factors for sepsis under treatment with eculizumab in patients with NMOSD. It seems reasonable, however, to exercise particular care when considering the use of eculizumab in patients with (a) acute infection (with an unresolved *Neisseria meningitidis* infection being an absolute contraindication); (b) a previous history of sepsis or, more generally, severe infections; (c) pre- or coexisting conditions that may cause immunosuppression (including connective tissue disorders [CTD], such as SLE, which are not uncommon in patients with AQP4-IgG-positive NMOSD); (d) conditions that are associated with a known increased risk for bacterial or fungal infections, including severe (NMOSD-related or other) disability resulting in decreased mobility (risk of pneumonia; risk of pressure ulcers with secondary sepsis), dysphagia (risk of aspiration pneumonia and secondary sepsis), bladder dysfunction (risk of urosepsis), or bowel dysfunction (ileus with secondary sepsis has been observed in a few patients treated with eculizumab); or (e) conditions that require additional immunosuppressive treatment (e.g., CTD). Last but not least, great care is required when considering the use of eculizumab together with immunosuppressive add-on therapies for NMOSD. Moreover, timely immunizations (mandatory against *Neisseria meningitidis* [ideally against serogroups A/C/W135/Y and B] in all patients and against *Streptococcus pneumoniae* and *Haemophilus influenzae* type b [Hib] infection at least in patients under the age of 18; further vaccinations according to national immunization guidelines recommended; checking vaccination titers recommended; booster vaccinations should be considered) are important, as is comprehensive instruction of patients about the increased risk of infection, about the early signs of meningitis, gonorrhea, sepsis, and infection in general, and about prophylactic measures [1]. It should be kept in mind (and patients informed) that vaccinations may not be effective in all cases [15]. In particular, our understanding of the effect of attack treatments and immunosuppressive therapy on vaccination success rates needs to be improved. Antimicrobial prophylaxis for the duration of eculizumab treatment has been discussed [15], but more studies are needed [5]. Finally, close monitoring for signs of infection and, if these are found, rapid and appropriate treatment is essential. Eculizumab treatment may need to be paused due to infection in selected cases but this bears a significant risk of disease exacerbation, which may occur soon after cessation (due to the drug’s short elimination half-life of around 11 days [1]) and requires particularly close monitoring. More data on the management of such patients are urgently needed.

In conclusion, (1.) TBIR needs to be considered in the differential diagnosis of patients with NMOSD and disturbed glucose homeostasis, especially, but not exclusively, in patients with co-existing SLE; (2.) while eculizumab (like the closely related drug ravulizumab) remains a highly effective treatment option for AQP4-IgG-positive NMOSD, the case described here highlights the risks associated with its use in patients who have autoimmune comorbidities that can compromise immune function and who may thus be at high risk of developing severe infectious complications. More data on the risk/benefit ratio of eculizumab in such patients are needed. Careful selection of patients for treatment with eculizumab, effective and timely immunization, and close monitoring for signs of infection in treated patients are essential.

## Abbreviations

AQP4: Aquaporin-4
AZA: Azathioprine
MMF: Mycophenolate mofetil
NMOSD: Neuromyelitis optica spectrum disorders
RTX: Rituximab
SLE: Systemic lupus erythematosus
TBIR: Type B insulin resistance
